# *In situ* tether formation from amines and alcohols enabling highly selective Tsuji–Trost allylation and olefin functionalization

**DOI:** 10.1039/c6sc04366f

**Published:** 2016-11-10

**Authors:** Ugo Orcel, Jérôme Waser

**Affiliations:** a Laboratory of Catalysis and Organic Synthesis , Ecole Polytechnique Fédérale de Lausanne , EPFL SB ISIC LCSO , BCH 4306 , 1015 Lausanne , Switzerland . Email: jerome.waser@epfl.ch

## Abstract

The use of tethers allows to overcome reactivity and selectivity issues often encountered with intermolecular reactions. Although tethers have been successfully applied for decades, their installation and removal usually requires additional steps. This minireview highlights the recent development of tethers that can be installed *in situ* on (homo)-allyl amines or alcohols for Tsuji–Trost allylation or double bond functionalization. In particular, the use of (hemi-)acetal tethers for highly regioselective and enantioselective Tsuji–Trost allylation was recently reported. Hydroamination of olefins starting from allylic amines could be achieved *via* a retro Cope-elimination using catalytic amount of an aldehyde for tether formation. Finally, bifunctionalizations of olefins were developed using either carbon dioxide or carbonyls/imines as tether precursors. These recent breakthroughs greatly enhanced the efficiency of the tethering approach for olefin functionalization, and will make it even more attractive for synthetic chemists in the future.

## Abstract 2

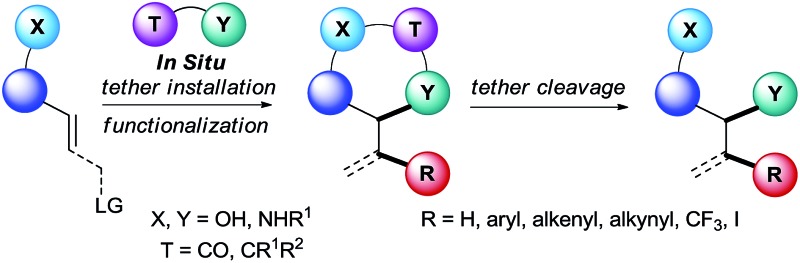
A new generation of methods based on *in situ* tether formation greatly enhances the efficiency and selectivity of olefin functionalization.

## 1. Introduction: the tethering approach for olefin functionalization

Olefins are key starting materials for the synthesis of valuable building blocks. Their broad availability as well as the extensive number of existing methods for their derivatization has made them essential in both academia and industry. Different strategies are commonly employed for the functionalization of either the α position or the double bond of alkenes. Intramolecular methods generally benefit from higher reactivity due to the lower entropies of activation. Furthermore, the regioselectivity and stereoselectivity can be more easily controlled based on the size and conformation of the formed ring. However, the substrates are often complex and accessed through long synthetic sequences. Furthermore, this strategy is limited to the synthesis of cyclic scaffolds with more narrow structural diversity, as normally it is difficult to change the innate regio- or stereo-selectivity induced by the ring size.

**Scheme 1:**
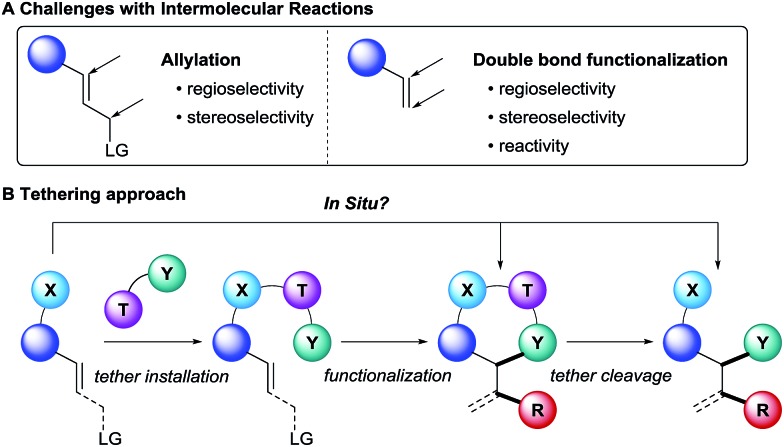
Challenges in intermolecular reactions and tethering approach.

Intermolecular reactions on the other hand are more versatile, since less functionalized and therefore readily available substrates can be employed. However, the transposition of known intramolecular reactions to fully intermolecular reactions can be a formidable challenge (Scheme 1A). Indeed, they are inherently more difficult to achieve since the high entropic cost to bring the reactants together lowers the reaction rate. Such energetic penalty can be overcome by using good leaving groups in the case of allylic functionalization, but it is particularly an issue when the modification of non-activated double bonds is considered. Furthermore, the stereo- and regioselectivity is more challenging to control because of the lack of cyclic transition state.

In this context, the use of tethers has emerged as an effective strategy to combine advantages of intra- and intermolecular reactions, such as simple starting materials, high reactivity and selectivity (Scheme 1B).^1^ Usually, the substrate has to bear a nucleophilic functionality, such as a hydroxy or an amino group, to attach the tether on it. Then the olefin functionalization step occurs in an intramolecular fashion. Finally, tether cleavage delivers the desired products. This approach requires the use of functionalized starting materials. When considering that many alcohols and amines in particular are broadly available from the biomass, this is not a strong limitation. As a consequence, the products synthesized *via* a tethering strategy are often highly functionalized.

Although tethers have been employed successfully for decades to address challenges of intermolecular reactions with alkenes, the multiple steps required to install and remove them have greatly limited their usefulness. Recently, new methods have emerged to circumvent this major drawback, in which the tethers are installed *in situ* onto alcohols or amines. This approach complements major progress realized recently in the use of removable or dynamic directing groups for the functionalization of olefins or allylation reactions.^2^ In order to achieve such performance, it is necessary to consider several criteria: (i) the tether installation must be fast and selective, (ii) excess of reagents and impurities should not impede the functionalization step, and (iii) final cleavage of the tether should be facile and as mild as possible.

**Fig. 1:**
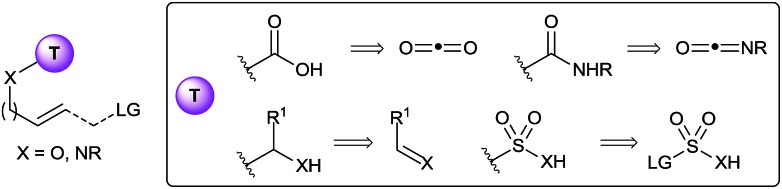
Commonly used tether and potential precursors for *in situ* formation.

Not surprisingly, researchers have focused on the *in situ* installation of well-established tethers (Fig. 1). For examples, carbonates and carbamates tethers have been intensively used for the synthesis of diols, aminoalcohols and diamines in both allylation reactions^3^ and the mono-^4^ and bi-functionalization^5^ of olefins. In principle, such tethers can be installed *in situ* by the use of either carbon dioxide or isocyanates as reagents. Prior to 2010, surprisingly few examples of such *in situ* tether formations had been reported. Using isocyanates, the palladium-catalyzed allylation for the synthesis of 1,2- or 1,3-oxazines was especially successful, starting either from epoxides/aziridines^6^ or carbonates^7^ as activated allyl alcohol equivalents. More rarely, the *in situ* formation of the carbamate directly from the free alcohol has also been applied.^8^ The use of carbon dioxide as tether precursor is even more challenging, and most success have been met for oxyhalogenation reactions.^9^ A disadvantage of the use of carbonyls as tethers is that cleavage can require relatively harsh reaction conditions. In this case, sp^3^ hybridized carbon tethers could be considered, as the obtained acetals, hemiaminals or aminals can be cleaved under milder conditions. Only rare examples of such tethers have been reported.^10^ Furthermore, *in situ* formation of the tether had never been achieved prior to 2010, even if aldehydes or imines would be potentially highly attractive precursors. Finally, heteroatom tethers, especially sulfonyl, have been also used highly successfully in the past, although again only using stepwise processes.^11^ Interestingly, a single example of the use of a boronate tether has also been reported by Cossy and co-workers in the Tsuji–Trost allylation.^12^

Recently, important progress has been realized for *in situ* tether formation, especially using sp^3^ hybridized carbon tethers obtained directly from the reaction of alcohols or amines with carbonyls or imines. This minireview will highlight selected recent examples which appeared since 2010. Progress with *in situ* tether formation in allylation reactions will be first presented, followed by the even more challenging mono- and bi-functionalization of the double bond of alkenes.

## 2. Tethers for regioselective Tsuji–Trost reactions

**Scheme 2:**
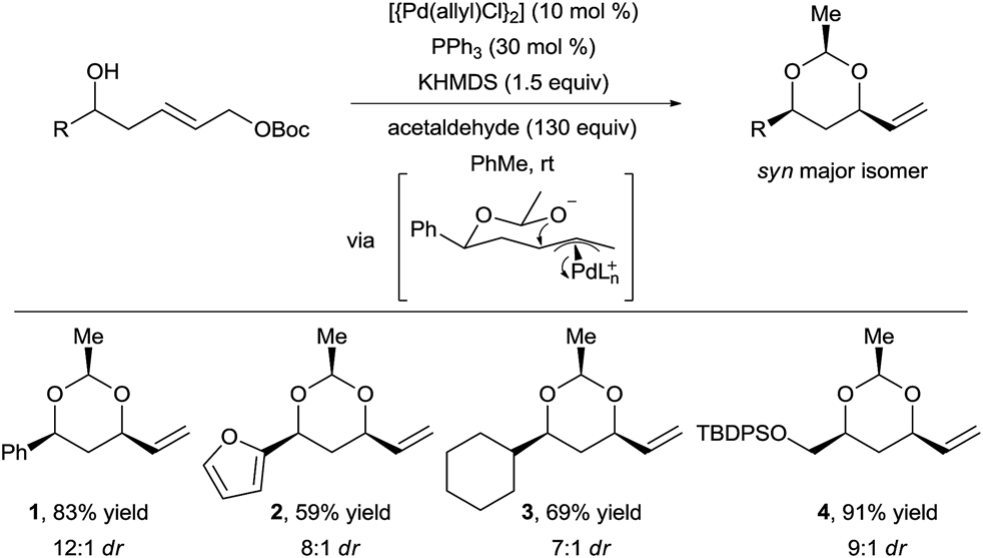
Domino hemiacetal formation–Tsuji–Trost reaction.

In 2012, Wang and Menche reported the use of acetaldehyde as tether for the synthesis of *syn* 1,3-diols *via* tandem hemiacetal formation and Tsuji–Trost reaction with a simple palladium catalyst (Scheme 2).^13^ This 3-step relay process involved (i) hemiacetal formation from a chiral homoallylic alcohols and acetaldehyde, (ii) palladium π-allyl complex formation, and (iii) intramolecular allylic substitution.

The homoallylic alcohol directs and controls both the regio- and stereoselectivity of the C–O bond formation *via* a cyclic chair transition state. Notably, the nucleophilic attack occurs on the internal position of the π-allyl intermediate, which would have been challenging to achieve in an intermolecular reaction. Arenes, heteroarenes, and functionalized alkyl chains were tolerated to give products **1–4** in good yield and diastereoselectivity. This strategy not only allowed expanding the scope of *O*-nucleophiles in the Tsuji–Trost reaction, but also delivering complex chiral 1,3-diols in a straightforward manner from simple substrates. In 2015, Aponick and co-workers reported a similar strategy but starting directly from non-activated allylic alcohols using either a gold(iii) or a bismuth(iii) catalyst.^14^

In 2013, Menche and co-workers reported the extension of the palladium-catalyzed methodology to the formation of 1,3-aminoalcohols.^15^ They employed *N*-tosyl aldimines as nitrogen source. However, all four possible diastereoisomers were formed during the reaction, with an overall low *syn*/*anti* ratio, which decreases its synthetic utility.

**Scheme 3:**
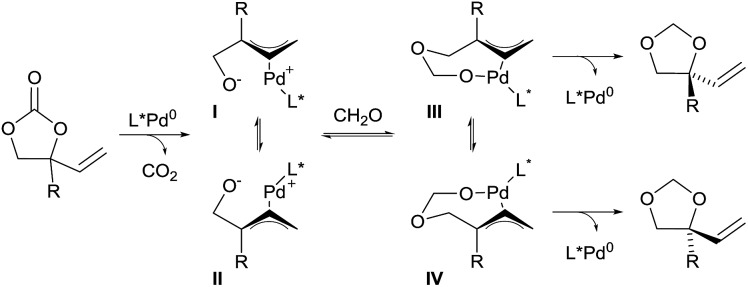
Domino decarboxylation–hemiacetal formation–Tsuji–Trost reaction.

In 2014, Zhang and co-workers also demonstrated the utility of aldehydes and aldimines tethers for the enantioselective synthesis of 1,2-diols and amino alcohols starting from racemic vinylethylene carbonates.^16^ Their strategy took advantage of a Pd-catalyzed decarboxylation, which afforded zwitterionic allylpalladium intermediates **I** and **II** with concomitant loss of the stereochemical information of the starting material through fast isomerization of the π-allyl intermediates (Scheme 3). Subsequent trapping of the alkoxide with a carbonyl affords intermediates **III** and **IV**. In presence of a chiral ligand, nucleophilic attack of one of the intermediates is favored to give 5-membered heterocycles with high regio- and enantio-selectivity.

**Scheme 4:**
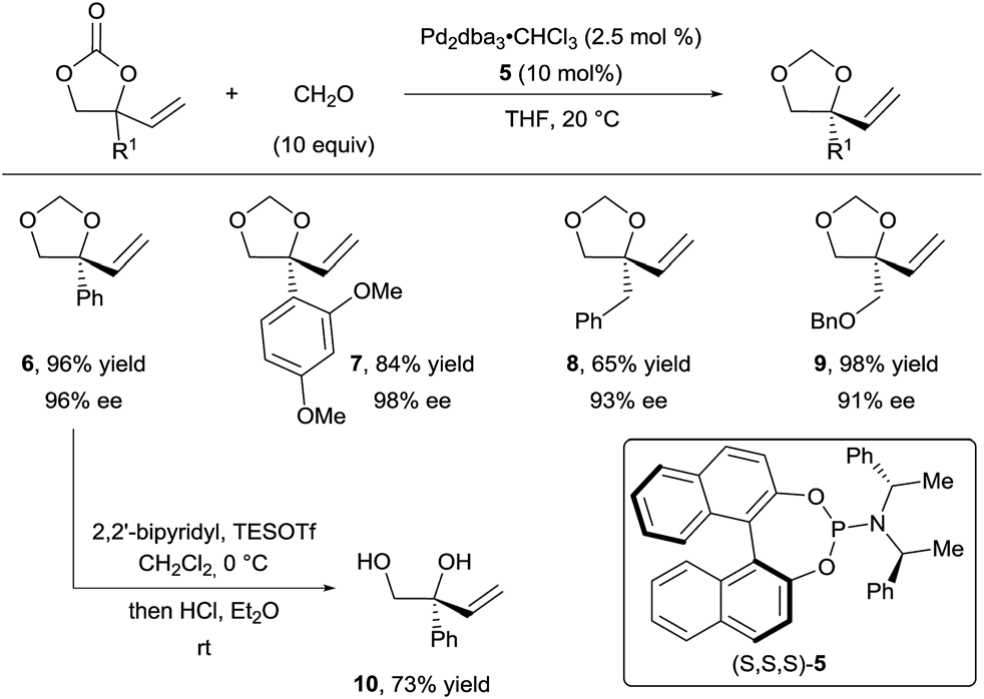
Access to chiral vicinal diols *via* decarboxylative Tsuji–Trost allylation.

In their first report, they described the use of an excess of formaldehyde to form the tether (Scheme 4).^16*a*^ They achieved excellent yields and enantioselectivities with a wide range of aryl (products **6** and **7**) and alkyl (products **8** and **9**) substituents using phosphoramidite ligand **5**. Free diols such as **10** could be obtained in good yields upon acidic treatment of the acetals. In addition, several aldehydes, such as acetaldehyde and benzaldehyde derivatives, were efficient for this transformation, both in terms of yield and enantioselectivity. However, the diastereoselectivity observed for the additional stereocenter was low.

**Scheme 5:**
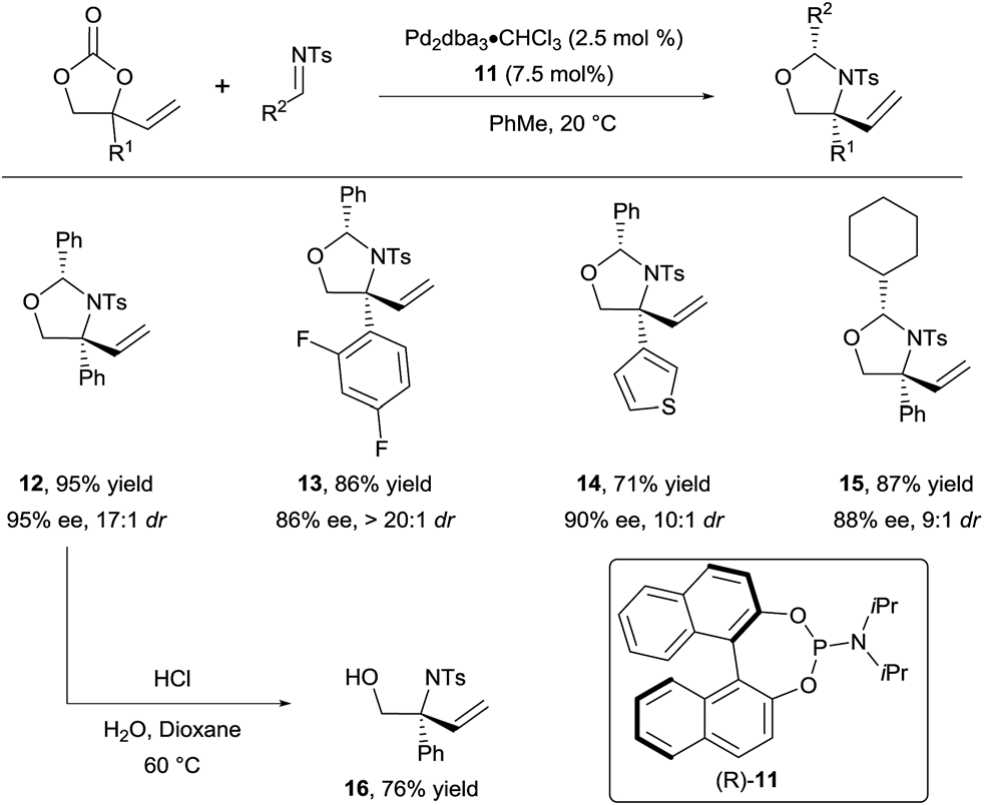
Access to chiral vicinal amino alcohols *via* decarboxylative Tsuji–Trost allylation.

In 2015, they successfully extended this method to the use of isocyanates^16*b*^ and aldimines^16*c*^ to form respectively oxazolidinones and oxazolidines. For the latter, a variety of *N*-tosyl aldimines could be engaged efficiently in the reaction (Scheme 5). Products **12–15** were obtained in high diastereoselectivity and enantioselectivity using phosphoramidite **11** as ligand. Importantly, the hemiaminal group could be readily cleaved under acidic conditions to unveil free amino alcohols such as **16**.

In summary, the Menche and the Zhang groups have demonstrated that tethers based on a sp^3^ hybridized carbon are highly efficient and practical for the synthesis of 1,2- and 1,3 amino alcohols and diols using the Tsuji–Trost allylation. High regio-, diastereo and enantio-selectivity could be achieved. Nevertheless, as olefins bearing a leaving group in α position are inherently activated substrates, it was not clear if such tethering strategies would also be successful in the more challenging functionalization of the π-bond of non-activated alkenes.

## 3. Tethers for olefin mono-functionalization

**Scheme 6:**
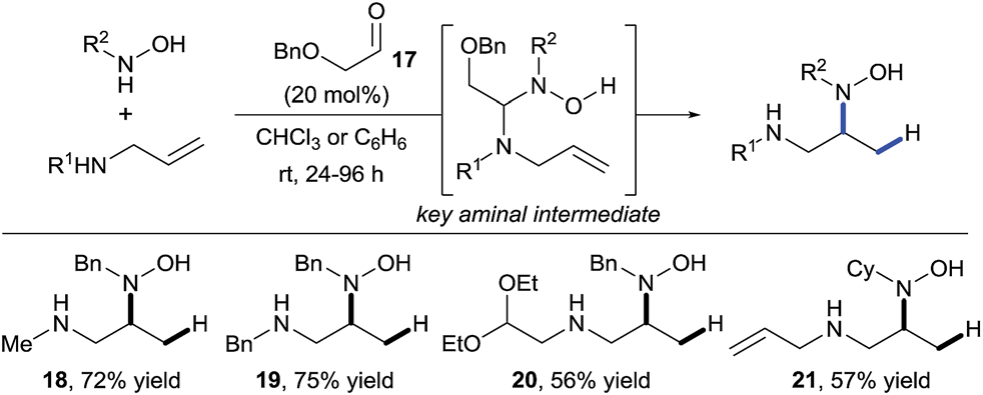
Diamine synthesis *via* retro-Cope elimination.

In 2011, building upon the work of Knight and coworkers on the stepwise retro Cope elimination,^17^ Beauchemin and co-workers reported the catalytic use of aldehydes as tether precursors for the metal-free synthesis of vicinal diamines.^18^ Their approach is based on a retro Cope elimination enabled by temporary intramolecularity. In their system, an allylic amine reacts with a nitrone generated *in situ* from an aldehyde and a hydroxylamine to deliver a key mixed aminal intermediate (Scheme 6). A Cope-type hydroamination of the olefin then occurs *via* a concerted, 5-membered cyclic transition state, leading to valuable 1,2-diamines products **18–21**. While several aldehydes could be used in stoichiometric amount, protected α-hydroxy aldehydes such as **17** were identified as efficient catalysts for this transformation. The electron withdrawing ether group activates the aldehyde and therefore facilitates the formation of the key aminal intermediate. The key features of this work are: (i) the reaction is metal-free, (ii) occurs at room temperature and (iii) the aldehyde can be used in catalytic amount. Hence, the tether is both installed and cleaved during the course of the reaction.

**Scheme 7:**
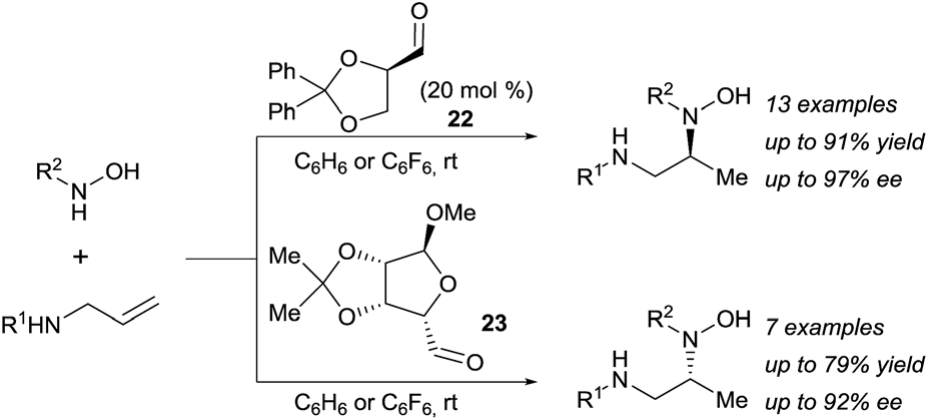
Catalytic asymmetric hydroamination *via* retro-Cope elimination.

The potential of this approach was further demonstrated with the development of a catalytic enantioselective version (Scheme 7).^18*c*^ Two readily available chiral aldehydes **22** and **23**, derived from glyceraldehyde and mannose respectively, were found to be efficient both in term of reactivity and enantiocontrol. Furthermore, they allowed the formation of both enantiomers. A limitation of this work was the need of a relatively high loading of aldehyde and the sensitivity of the reaction towards steric hindrance. Indeed, amines bearing a bulky substituted at their allylic position and also internal olefins were poorly reactive under the developed conditions.

**Scheme 8:**
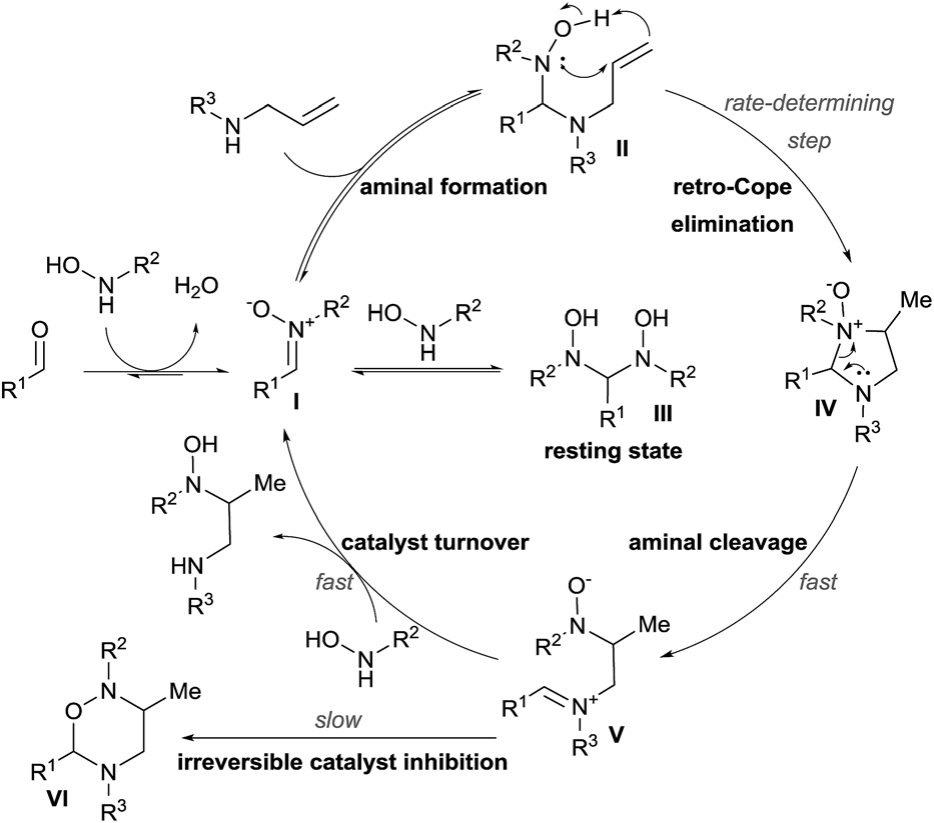
Catalytic cycle of the Cope-type hydroamination.

Mechanistic studies were then conducted, leading to a first proposal for the catalytic cycle (Scheme 8).^18*b*^ The reaction starts with condensation of the hydroxylamine with the aldehyde to give nitrone **I**. Formation of aminal **II** followed by retro-Cope elimination leads then to cyclic aminal **IV**. C–N bond cleavage then gives iminium **V**. The catalytic cycle is completed by reaction with the hydroxylamine to free the hydroamination product.

Kinetic experiments revealed that the reaction was first order both in the allylamine and the aldehyde catalyst, but inverse first order in the hydroxylamine. The latter is explained by the formation of the non-productive aminal **III** by condensation of two molecules of hydroxylamine on the aldehyde. Inhibition of the catalyst occurs *via* the formation of oxadiazinanes **VI**, which were the products described by Knight and coworkers.^17^ The rate-determining step was established to be the retro-Cope elimination and the rate law of the reaction involved the formation of the aminal, as it occurs before this step.

These important findings enabled them to re-design their catalyst. Initial attempts to lower the activation energy for the hydroamination step were not fruitful, as the equilibrium to form **II** was more important. In particular, this step was very sensitive to steric effects and therefore formaldehyde was the best catalyst. Interestingly, the reaction could be performed using formalin in aqueous solution. The catalyst loading could be lowered to 5 mol% without erosion of the catalytic activity. The scope of the reaction could be expanded to branched allylamines, which delivered the corresponding diamines in high diastereoselectivity and good yields. Less reactive internal alkenes could also be successfully engaged in the reaction.^18*d*^

## 4. Tethers for olefin difunctionalization

**Scheme 9:**
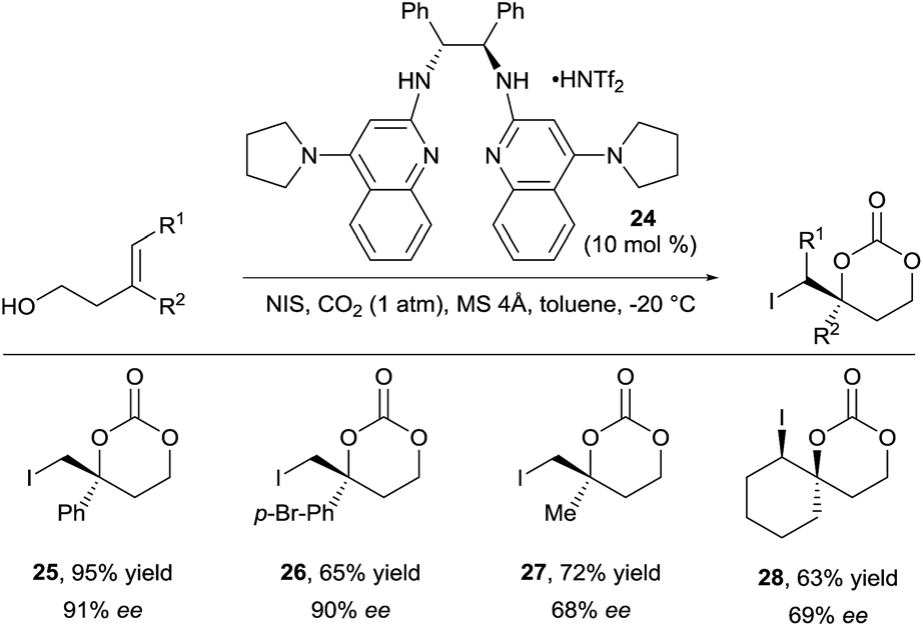
Enantioselective carbonate synthesis with carbon dioxide.

For a long time, difunctionalization of olefins *via in situ* tether formation has been limited to oxy- or amino-halogenation reactions using carbon dioxide.^9^ The obtained products are highly attractive for further functionalization and research in this area is therefore still ongoing. A highly efficient method for the oxy- and amino-iodation of both allylic alcohols and amines was developed by Minakata and co-workers using *tert*-butyl hypoiodite as iodine source with only one atmosphere of carbon dioxide.^19^ An important breakthrough was reported by Johnston and co-workers in 2015 with the development of the first enantioselective oxy-iodation using carbon dioxide for tether formation (Scheme 9).^20^ By using bisamidine catalyst **24** and *N*-iodo succinimide (NIS) as iodine source, benzylic carbonates **25** and **26** were obtained in high yield and enantioselectivity. Moderate enantioselectivity was obtained in case of aliphatic or trisubstituted olefins (products **27** and **28** respectively).

**Scheme 10:**
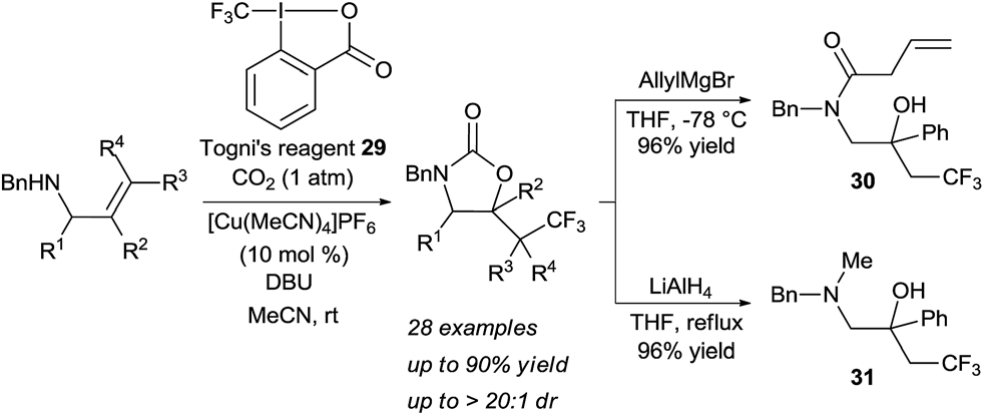
Oxytrifluoromethylation of allylamines using carbon dioxide.

Transformations beyond halogenations using carbon dioxide were limited for a long time to activated systems, such as allenes.^21^ Recently Yu and coworkers disclosed a Cu-catalyzed oxy-trifluoromethylation of allylamines using CO_2_ gas to form the tether, in which one C–O and one C–C bonds are formed (Scheme 10).^22^ The combination of a nucleophilic allylic amine, CO_2_ and Togni's reagent **29** in presence of a copper catalyst and a base led to the oxy-trifluoromethylation of the olefin in high yield and diastereoselectivity. Notably, the developed reaction allowed for mild conditions with CO_2_ at atmospheric pressure. Cleavage of the heterocycle was achieved either with the strong reducing agent LiAlH_4_ or allyl magnesium bromide to afford the corresponding vicinal amino alcohols such as **30** and **31** in high yield.

**Scheme 11:**
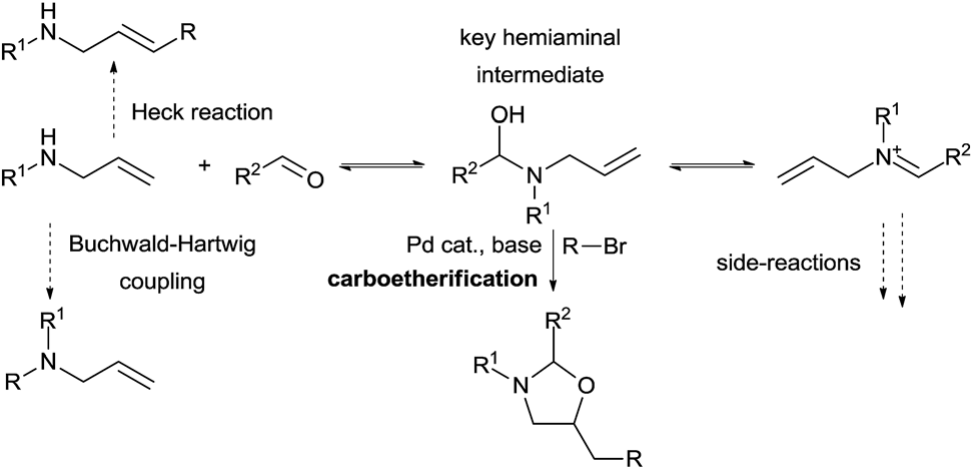
Vicinal amino alcohol synthesis *via in situ* aminal formation.

The use of other tether precursors beside CO_2_ has been less investigated. Inspired by the work of Beauchemin and co-workers involving aminal intermediates, our group aimed to develop a similar strategy for more complex olefin difunctionalization using a palladium catalyst (Scheme 11). In a first step we hypothesized that a simple allylamine and an aldehyde could *in situ* form a hemiaminal that could then undergo a palladium-catalyzed carboetherification reaction with an adequate electrophile. Many possible side reactions had to be prevented to make this process feasible. Quantitative formation of the tether is necessary to prevent direct Heck reactions, Buchwald-Hartwig coupling, or catalyst inhibition by the amine. Therefore, hemiaminal formation should be both kinetically rapid and thermodynamically favored over both the allylamine and the iminium, as the latter can lead to the formation of undesired aminals. Furthermore, the key hemiaminal intermediate must engage efficiently in the carbo-oxygenation reaction.

**Scheme 12:**
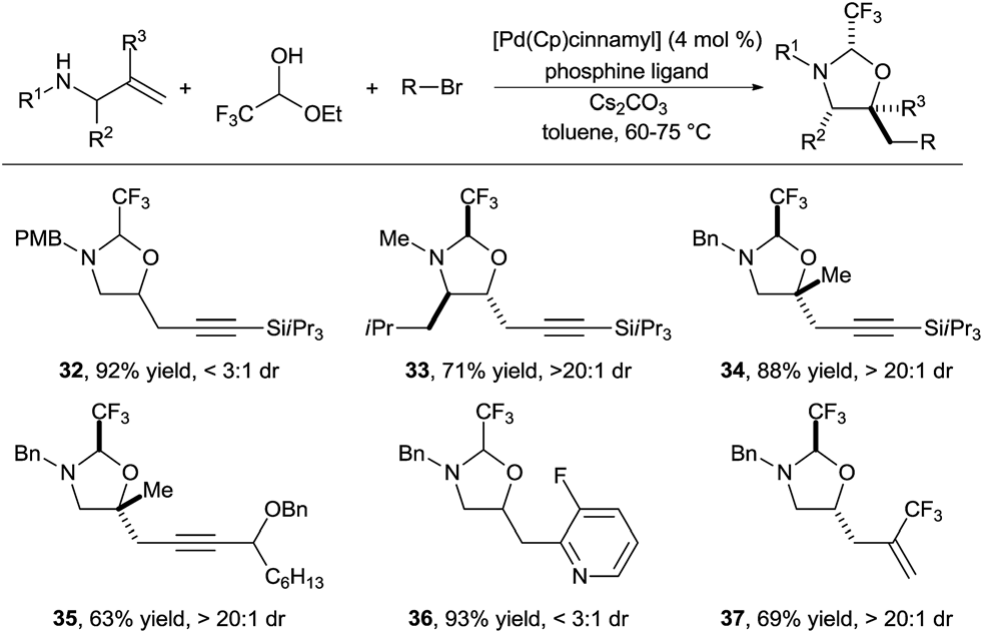
Vicinal amino alcohols synthesis *via* formation of imidazolidines.

In 2015, we reported the first implementation of this strategy for the synthesis of amino alcohols (Scheme 12).^23^ The use of the highly electron deficient trifluoroacetaldehyde in its hemiacetal form in combination with electron-rich allylamines was essential to obtain high reactivity. This method allowed the introduction of alkynyl, aryl and vinyl groups in good to excellent yields (products **32–37**). Depending of the substrate class, either biphosphines (such as DPEPhos or XANTPhos) or the monodentate trifurylphosphine were the best ligand on palladium. The reaction developed was completely regioselective, whereas the stereoselectivity was substrate dependent. Nevertheless, control of the stereochemistry at the hemiaminal center is less important, as the tether can be cleaved easily. In the case of α-substituted allylamines, the existing stereocenter controlled efficiently the formation of the two others stereocenters (product **33**). Tertiary ether centers could be also formed in good yield (products **34** and **35**).

**Scheme 13:**
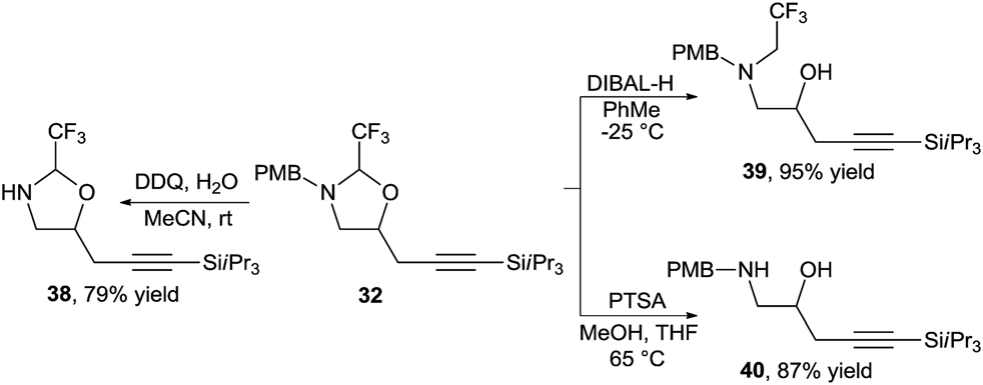
Orthogonal deprotection of oxazolidines.

The oxazolidines could be easily transformed into other useful building blocks (Scheme 13). Orthogonal deprotection was possible to access either free amine **38** or free alcohol **39**. The hemiaminal tether could be readily removed under acidic conditions to afford amino alcohol **40** in high yield.

**Scheme 14:**
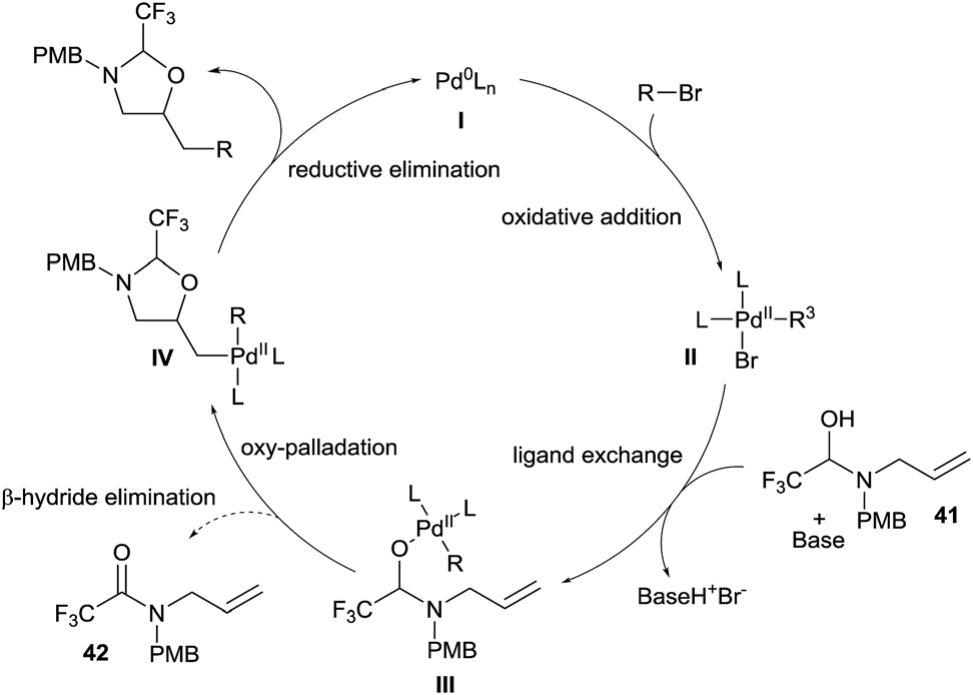
Speculative mechanism for the synthesis of aminoalcohols *via in situ* tether formation.

The speculative mechanism of this reaction, based on studies in our group and others,^24^ would start with the oxidative addition of an organohalide on a Pd(0) complex **I** to form complex **II** (Scheme 14). Base mediated ligand exchange on **II** with hemiaminal **41** would generate the Pd(ii)-alkoxide **III**. At this point, either the productive key oxypalladation occurs to form intermediate **IV**, or β-hydride elimination is competitive and produces amide **42**.^25^ Finally, reductive elimination allows the formation of the desired oxazolidine and re-generates the palladium(0) complex **I**.

**Scheme 15:**
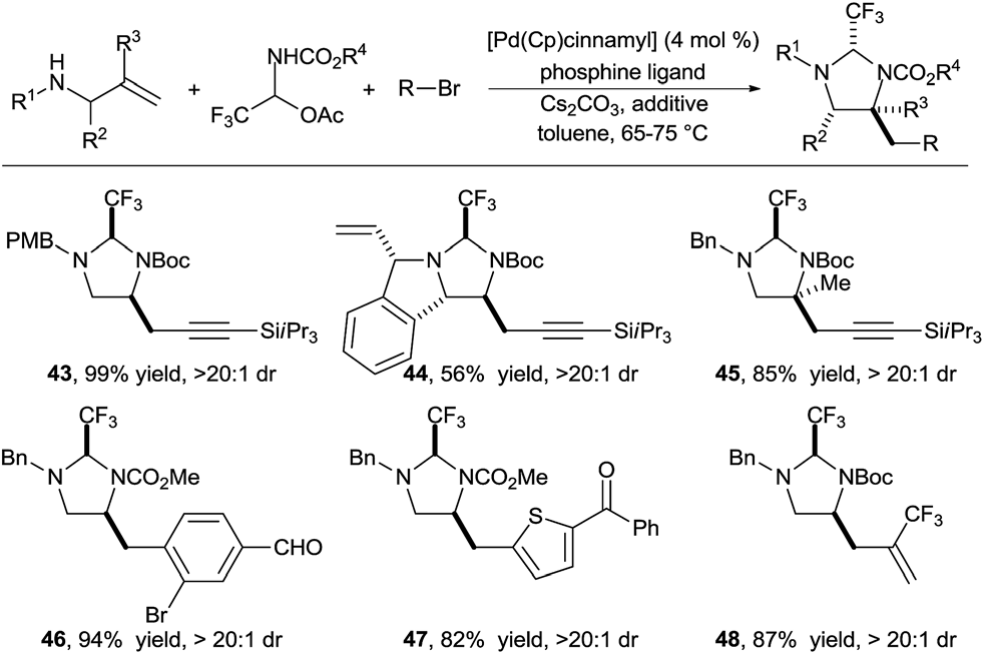
Vicinal diamine synthesis *via* formation of imidazolidines.

In 2016, we reported the extension of this strategy to the synthesis of diamines (Scheme 15).^26^ We identified carbamate *N*-protected trifluoroaldimines in their stable hemiaminal form as tether precursors of choice for this transformation. The trifluoromethyl group was again essential to ensure the fast formation of a very stable aminal, as well as full conversion for the Pd-catalyzed carboamination. This practical procedure allowed to efficiently access a wide range of functionalized diamines. A large variety of alkynyl, aryl and hetereoaryl groups could be introduced in high yield under mild conditions (products **43–48**).

**Scheme 16:**
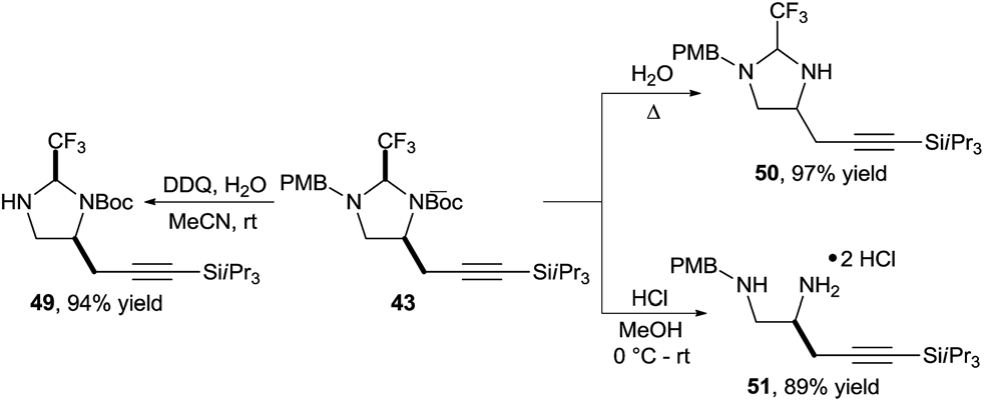
Orthogonal deprotection of imidazolidine **43**.

Also in this case, the obtained imidazolidines could be easily modified (Scheme 16). Selective Boc or PMB deprotection was possible to give amines **49** and **50** respectively. Alternatively, diamine **51** could be obtained under mild acidic conditions.

Our work consequently established the use of tethers derived from trifluoroacetaldehyde for the bifunctionalization of allyl amines. This type of tethers is expected to find many more application in the future, not only for olefin functionalization, but also for other types of transformations.

## 5. Conclusion and outlook

Tethering strategies have been used since a long time for the functionalization of olefins and in the Tsuji–Trost allylation reaction to overcome limitations of reactivity and selectivity inherent to intermolecular processes. Nevertheless, this approach traditionally involves multi-step procedures for tether installation and removal, which makes it synthetically less useful. Recent progress for *in situ* tether installation has begun to address this shortcoming, leading to an important increase in synthetic efficiency without jeopardizing selectivity and enhanced reactivity. The first example of catalytic tether formation, albeit for the moment limited to metal-free transformations, constitutes an even more efficient approach, and paves the way for future impressive breakthroughs.
